# Impact of sample multiplexing on detection of bacteria and antimicrobial resistance genes in pig microbiomes using long-read sequencing

**DOI:** 10.3389/fmicb.2025.1597804

**Published:** 2025-06-19

**Authors:** Mirena Ivanova, Frank M. Aarestrup, Saria Otani

**Affiliations:** Research Group for Genomic Epidemiology, National Food Institute, Technical University of Denmark, Kgs Lyngby, Denmark

**Keywords:** microbiome, long read sequencing, AMR, bacteria, multiplexing

## Abstract

The effects of sample multiplexing on the detection sensitivity of antimicrobial resistance genes (ARGs) and pathogenic bacteria in metagenomic sequencing remain underexplored in newer sequencing technologies such as Oxford Nanopore Technologies (ONT), despite its critical importance for surveillance applications. Here, we evaluate how different multiplexing levels (four and eight samples per flowcell) on two ONT platforms, GridION and PromethION, influence the detection of ARGs, bacterial taxa and pathogens. While overall resistome and bacterial community profiles remained comparable across multiplexing levels, ARG detection was more comprehensive in the four-plex setting with low-abundance genes. Similarly, pathogen detection was more sensitive in the four-plex, identifying a broader range of low abundant bacterial taxa compared to the eight-plex. However, triplicate sequencing of the same microbiomes revealed that these differences were primarily due to sequencing variability rather than multiplexing itself, as similar inconsistencies were observed across replicates. Given that eight-plex sequencing is more cost-effective while still capturing the overall resistome and bacterial community composition, it may be the preferred option for general surveillance. Lower multiplexing levels may be advantageous for applications requiring enhanced sensitivity, such as detailed pathogen research. These findings highlight the trade-off between multiplexing efficiency, sequencing depth, and cost in metagenomic studies.

## Introduction

Advancements in sequencing technologies have markedly improved metagenomics and microbiome research, allowing for accurate and comprehensive analysis of complex microbial communities (e.g., Satam et al., 2023). Short-read sequencing platforms, such as Illumina, have been widely used for metagenomic studies, including pathogen surveillance and antimicrobial resistance, due to their high accuracy, throughput and cost-effectiveness (Quince et al., 2017; Hendriksen et al., 2019; Bogri et al., 2024; Kohle et al., 2024; Royer et al., 2024). However, they also result in fragmented contigs and genomes, and might suffer to resolve complex or repetitive genomic regions, especially in microbial communities with a high bacterial diversity, closely-related species/strains, or a rich antimicrobial resistance gene (ARG) reservoir (Albertsen et al., 2013; Quince et al., 2017; Sereika et al., 2022). Illumina-based research has also been conducted over a longer period of time compared to newer sequencing technologies (Goodwin et al., 2016; Satam et al., 2023), thus its performance, such as library preparation, platform, depth, coverage, and the impact of multiplexing, has been studied relatively more (Campbell et al., 2015; Poulsen et al., 2022; Ribarska et al., 2022). In contrast, the effect of sequencing output, particularly in terms of multiplexing and data yields, remains unexplored for newer long-read technologies such as Oxford Nanopore Technologies (ONT).

The introduction of ONT platforms, such as GridION and PromethION, provide increased resolution of microbial communities, ARGs, and pathogenic species (Meslier et al., 2022; Agustinho et al., 2024). Because of their longer reads, they result in more complete metagenome-assembled genomes (MAGs), and with increased N50 that varies for different DNA extraction methods and metagenomic sample types, e.g., from 4 kb (Latorre-Pérez et al., 2020) to up to 10–15 kb reads (Buttler and Drown, 2022). This capability is particularly valuable for applications such as metagenomic comparison and surveillance of closely-related species and antimicrobial resistance (AMR) genes, as short reads may have challenges capturing the critical DNA regions for species or gene differentiation. Longer DNA sequences provide better coverage of these regions and may help resolve bacterial or gene assignment issues. Importantly, the ability to accurately resolve microbiome composition is also dependent on sequencing output, as a higher number of reads provides more comprehensive information on the composition and functional potentials in complex microbial communities (Sharon et al., 2015; Liao et al., 2023). In any sequencing technology, the number of reads per sample is directly influenced by the number of samples sequenced on a single flowcell, also known as multiplexing.

The optimal use of any ONT flowcell capacity, especially with various sample multiplexing, remains largely underexplored. Particularly how the number of samples multiplexed per flowcell affects detection sensitivity, and data quality and size, to resolve microbial and ARG diversities. This is especially relevant in the context of surveillance programs, where sequencing costs and throughput must be balanced with the need for accurate detection of resistance genes and clinical infectious agents in a short time (Besser et al., 2018; Liu et al., 2021a; Kumburu et al., 2023). To date and to the best of our knowledge, there has been no comparative analysis of sample multiplexing between the different ONT platforms, GridION and PromethION, and how varying multiplexing levels may affect the sensitivity of ARG and bacterial detection. PromethION platform with its higher data output capacity compared to GridION (Latorre-Pérez et al., 2020) may allow for greater multiplexing, but it remains unclear whether or how increased sample numbers per flowcell might lead to decreased sensitivity, particularly for low-abundance species and ARGs.

In this study, we assessed the impact of sample multiplexing on the detection sensitivity of ARGs and bacterial taxa using two long read ONT sequencing platforms: GridION and PromethION. Specifically, we compared bacterial and ARG communities in the same pig microbiome multiplexed at two levels (four and eight samples) on both platforms. We also evaluated the ability of both platforms to detect representatives of pathogenic bacterial species across all multiplexed samples.

## Materials and methods

### Experimental design

**Figure 1:**
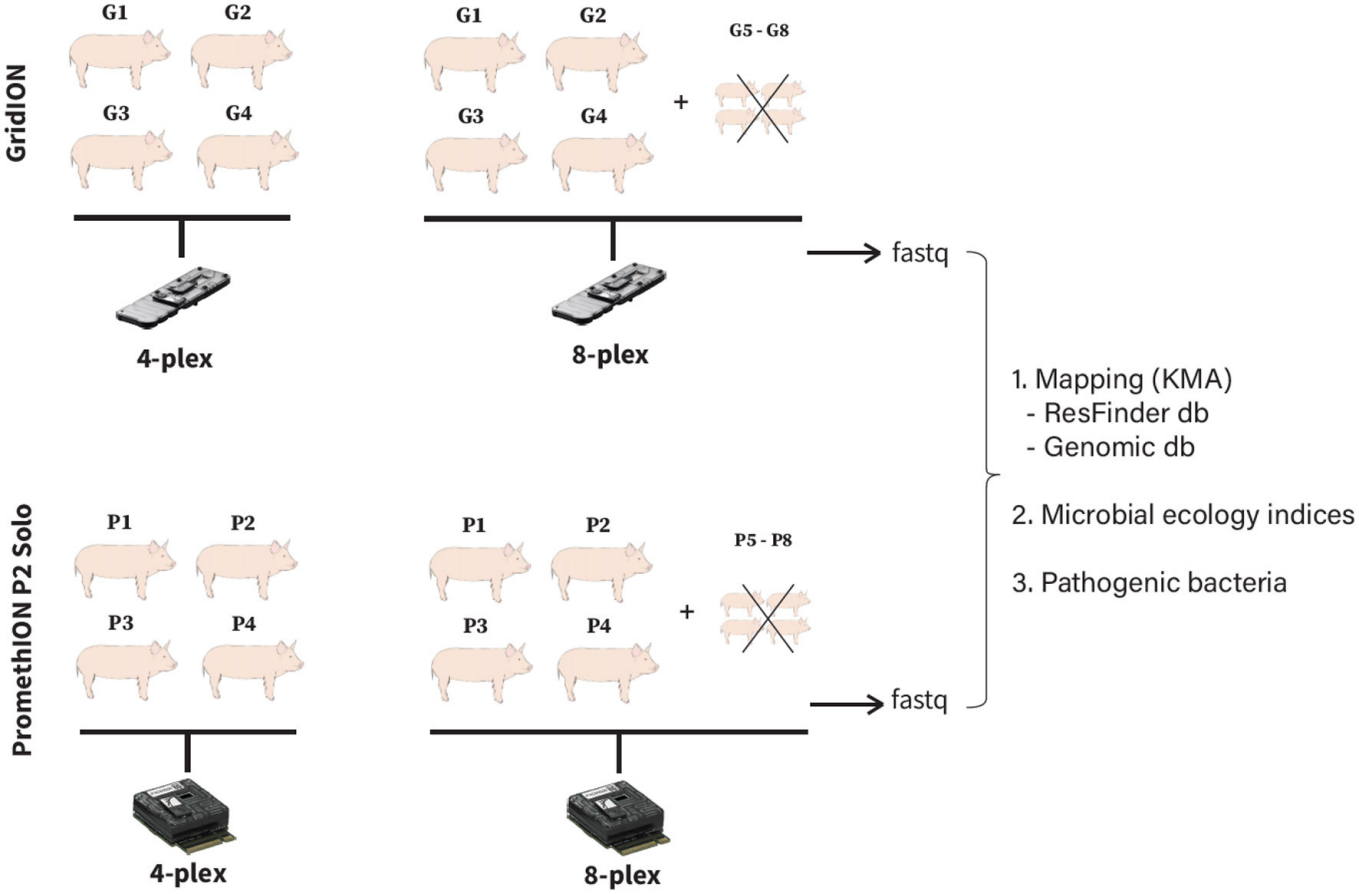
Study experimental design. G1-G4: pig fecal microbiomes sequenced on GridION at four- and eight-plex; P1–P4: pig fecal microbiomes sequenced on PromethION at four- and eight-plex.

A total of four different pig fecal samples were selected for this study (Figure 1). They were sequenced as four and eight samples per flowcell on GridION, and four and eight samples per flowcell on PromethION (P2 Solo version) (Figure 1). We only tested four and eight samples per flowcell as running one sample per GridION or PromethION flowcell would be under-utilizing the system capacity. To confirm this, we have tested single-plex sample on GridION as it theoretically produces max 48 Gb (ONT: https://nanoporetech.com/products/sequence/gridion) and confirmed it would be under-utilized (details in Supplementary material), therefore all our downstream analyses is based on four- and eight-plexed metagenomics. In all eight-plex flowcells, four additional fecal samples were included solely to fill the flowcell capacity to eight. These extra samples were not part of the four-sample set also sequenced in the four-plex runs, and were therefore excluded from downstream analysis to ensure that comparisons between four- and eight-plex conditions were based on the same biological samples (Figure 1). To assess whether the variation in ARG and bacterial detections was due to multiplexing levels or simply the inherent variability in sequencing replicates, we performed additional triplicate sequencing of the same samples under four- and eight-plex conditions on both GridION and PromethION for all the sequencing runs. This allowed us to determine whether the detected differences in ARG and bacterial species were attributed to multiplexing rather than sample-to-sample sequencing variability. All fecal samples originated from Danish healthy pigs submitted to the Danish Integrated Antimicrobial Resistance Monitoring and Research Programme (DANMAP https://www.danmap.org/) (Aarestrup et al., 1998). All pig fecal samples were selected from geographically close farms with very similar production settings and feeding regimes. Additionally, the pigs were all of the same age and similar weight, minimizing biological variability. The samples were stored at −80°C immediately after collection, thawed on ice and 170 (± 5) mg were weighted and used for subsequent DNA extraction.

### DNA extraction, library preparation and ONT sequencing

Total DNA was extracted using the Quick-DNA HMW Magbead Kit (Cat. No. D6060, Zymo Research) following the manufacturer's instruction with minor modifications: 170 ± 5 g feces were suspended in increased volume of 200 uL DNA/RNA shield R1100, followed by incubation for 10 min—during microbial lysis, treatment was done with larger volume of 100 uL lysozyme (100 mg/ml) and the incubation during DNA purification was prolonged to 15 min. DNA yield was measured using the Qubit 4 Fluorometer (Cat. No. Q33238, Invitrogen), and was stored at 4°C until library preparation. From each sample, 1 μg DNA input was used for library preparation with the same Ligation gDNA Native Barcoding Kit 24 V14 (SQK-NBD114.24, Oxford Nanopore Technologies, Oxford) following the manufacturer's instruction with minor modifications: increased incubation times during the end prep. to 10 min, and during the barcode and adaptor ligation steps to 40 min. Four and eight samples were multiplexed and loaded on FLO-PRO114M flowcell (R10.4 M chemistry) and sequenced on PromethION P2 Solo platform, and the same four and eight samples were multiplexed and loaded on FLO-MIN114 flowcell (R10.4 chemistry) and sequenced on GridION platform. Sequencing was performed for 72 h and reads were basecalled using GuppyBasecaller (v7.2.13) with super-accurate basecalling option. Low-quality reads were filtered using the default setting on MinKNOW with reads below a score of < 9 and read lengths < 200 bp to be omitted.

### ARGs and bacterial assignment and data analysis

Taxonomic and gene assignment of the reads was carried out as described in Bogri et al. (2024). Briefly, raw sequence data were mapped with KMA v1.4.12a (Clausen et al., 2018) against a custom reference genomic database (last updated 22/05/2024) that was used previously for taxa assignments (Hendriksen et al., 2019; Jensen et al., 2023), comprising of NCBI GenBank databases of bacteria (closed genomes), archaea, MetaHitAssembly (PRJEB674–PRJEB1046), HumanMicrobiome (genome assemblies), and bacteria_draft.

For ARG assignment, all reads were aligned with KMA v1.4.12a to the ResFinder database v4.0 (last updated 14/05/2024), consisting of known and acquired resistance genes (Zankari et al., 2017; Bortolaia et al., 2020).

To calculate the abundance of bacteria and ARGs from long read sequencing data, unlike conventional approaches where read count is used as a parameter in Illumina sequencing where read lengths are all equals (e.g., Jensen et al., 2023), we adapted a depth calculation approach that is specific to ONT, accounting for its long and variable read lengths. This approach allows for more accurate taxa and gene abundance estimates as it avoids calculating abundance based on read counts (which varies in number and length in long read sequencing compared to Illumina), and uses basepair-based approach, which takes into account the entire sequencing run output regardless of the read counts (whether short or long). This approach takes into account how many reads were assigned to a taxon or gene, in addition to the length of those reads as an input since the reads vary in length. The depth approach was determined for each feature as follows: First, each ARG or bacterial taxon was considered present in a sample if its coverage was ≥0.9 when aligned to its reference in the database to increase our confidence for calling the ARGs or bacterial taxa. Second, the coverage of each ARG or bacterial species in a sample was calculated by dividing the number of covered reference basepairs (namely refCoveredPositions in KMA.mapstat output file) by the basepair length of each ARG or bacterial genome in the reference database. Lastly, the total basepairs aligned to each detected ARG or bacterial species were divided by the total length of all reference ARGs or bacterial species, respectively, that met the ≥0.9 coverage threshold and were found in that sample. All those depth values were then log10-transformed when visualizing in the downstream analyses. Python scripts are made available on GitHub (https://github.com/mirivan2/create_files_from_mapstat) to calculate ONT depths using this approach. The calculated feature depths were further used for comparison between samples sequenced on GridION and PromethION or between the sequenced triplicates.

Simpson and Shannon diversity indices (Simpson, 1949; Ortiz-Burgos, 2016), and the Chao1 richness estimate (Chao, 1984) were calculated at species taxonomic levels and for all identified ARGs using the diversity function in the “vegan” R package v2.6.6.1 (Oksanen, 2024) and visualized using “ggplot2” R package v3.5.0 (Wickham, 2016). Heatmaps were generated using the “pheatmap” R package v.1.0.12. Kruskal–Wallis test with Benjamini-Hochberg (BH) correction (adjusted *p-*value < 0.05) was used to test for statistical significance between the detected ARGs and bacteria at four- and eight-plex for GridION and Promethion (Kruskal and Wallis, 1952). In addition, the ALDEx2 tool based on Welch's *t-*test and multiple testing correction using the BH procedure was performed to control for false discovery rate (FDR) (Gloor et al., 2016). Beta diversity represented by a principal component analysis (PCA) of Bray-Curtis dissimilarities between four- and eight-plexed samples was performed by “vegan” and visualized by “ggplot2”.

## Results and discussion

### Comparison of the sequencing outputs between GridION and PromethION across different multiplexing levels

Since ONT produces reads of varying lengths, we used basepair depth comparisons to more accurately reflect sequencing output, rather than read counts as typically done in Illumina sequencing. We therefore use only basepair output when comparing the sequencing output of fecal microbiomes from four pigs, sequenced as four-plex and eight-plex on both GridION and PromethION (Figure 1), in all downstream analyses below.

**Figure 2:**
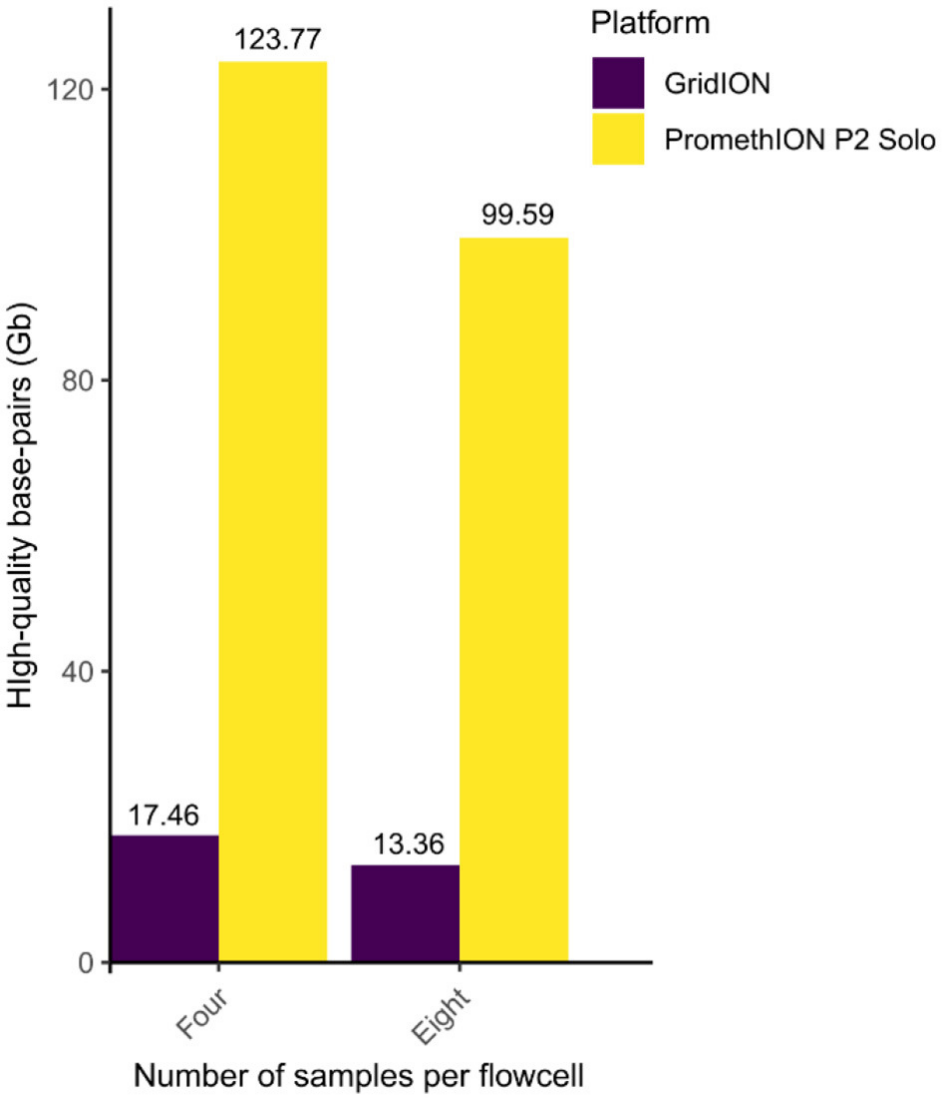
Total number of high quality basepairs for four and eight samples per flowcell sequenced on GridION (purple) and PromethION (yellow). Number of individual sample outputs and high quality reads are in Supplementary Figure S1.

From PromethION, the total number of high quality basepairs (quality *Q*-score of ≥10) were 123.77 Gb in the four-plex setting (from all four samples combined; Figures 1, 2; Supplementary Figure S1), which decreased to 109.64 Gb in the eight-plex (from all eight samples combined; Figure 2; Supplementary Figure S1). Similarly, for GridION, the high-quality base pairs decreased from 17.46 Gb in the four-plex to 13.36 Gb in the eight-plex flowcell (Figure 2; Supplementary Figure S1). On average, for 72-h sequencing PromethION produced approximately 2.1X more basepairs per sample in four-plex (30.94 ± 6.87 Gb) than in eight-plex (13.98 ± 3.44 Gb) (Supplementary Figure S1; Supplementary Figure S2), which should be compared to the expected 2-fold reduction. On GridION, sequencing output varied less between the four- and eight-plex runs over the same sequencing time, compared to PromethION (Supplementary Figures S1, S2), likely due to differences in platform throughput capacity, with PromethION designed for substantially higher data yields per flowcell (Latorre-Pérez et al., 2020; Oxford Nanopore Technologies, 2024). Overall, PromethION generated up to nine times more high-quality basepairs than GridION (Supplementary Table S1). Despite that both platforms and multiplexing levels used comparable DNA input (~1 μg) and the same library preparation method over the same sequencing period. This difference can be attributed to the larger capacity of the PromethION FLO-PRO114M flowcell compared to the GridION FLO-MIN114 flowcell. Theoretically, PromethION flowcells can yield up to 290 Gb per flowcell over 72 h when using R10.4.1 chemistry for metagenomic samples [Oxford Nanopore Technology https://nanoporetech.com/document/requirements/promethion-2s-spec, (Fernandes et al., 2014; Latorre-Pérez et al., 2020)]. The four-plex generated more data per sample compared to eight-plex on both platforms (Supplementary Table S1), which is expected as less sample on a flowcell will allow higher throughput per sample.

The total number of reads and basepairs produced by GridION and PromethION at four- and eight-plex for the four samples, as well as the number of reads mapped to ARGs and bacterial species, are in Supplementary Table S1.

### Resistome variations across multiplexing levels between microbiomes on GridION and PromethION

**Figure 3:**
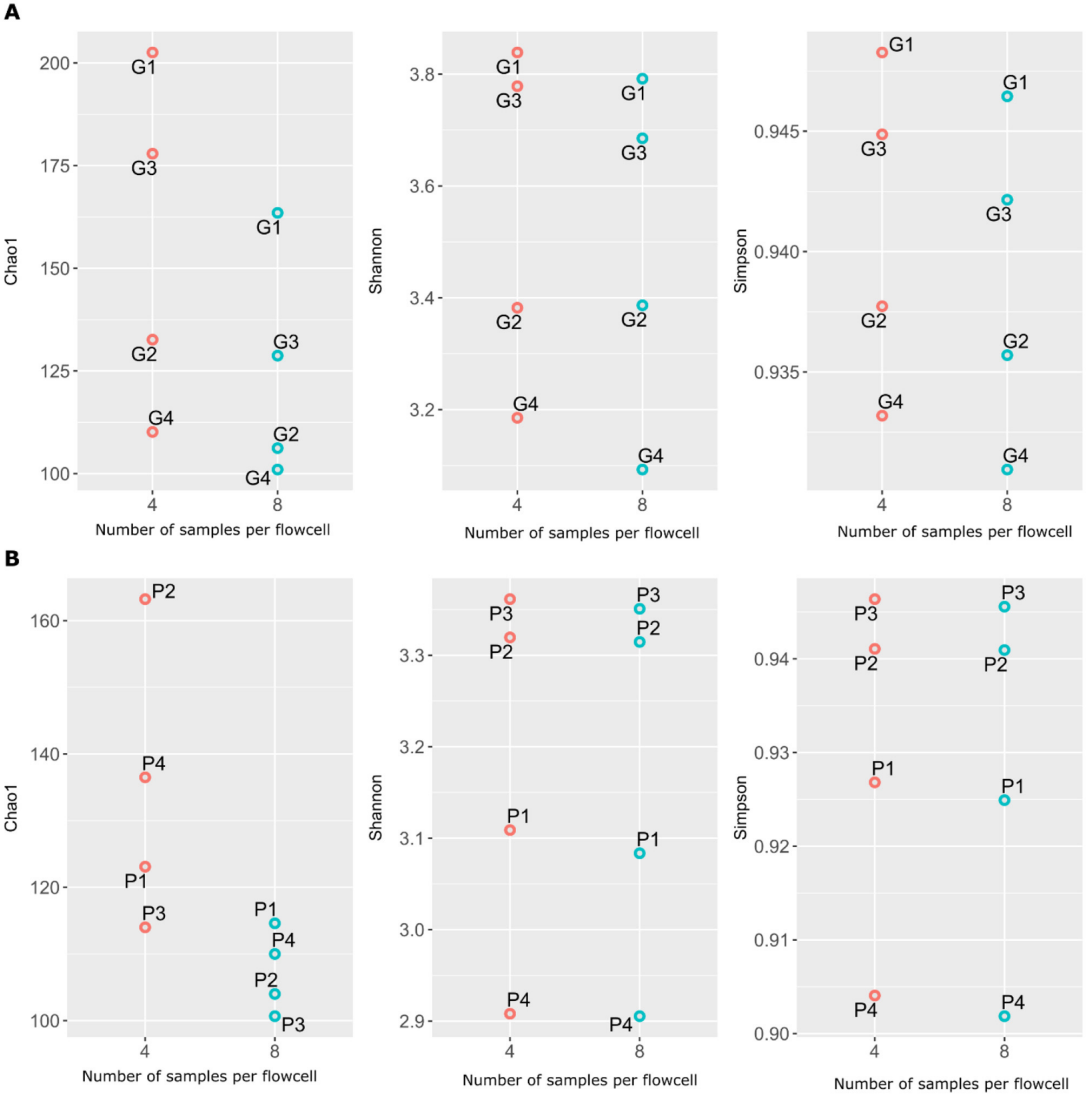
Variations in ARGs richness and diversity with four- and eight-plexed microbiomes on GridION **(A)** and PromethION P2 Solo **(B)**.Red circles are four-plex, blue circles are eight-plex.

Overall, resistome richness (Chao1) decreased with increasing multiplexing on both GridION and PromethION, while resistome diversity (Shannon and Simpson indices) remained relatively stable across different multiplexing levels (Figure 3). This trend was consistent across both platforms. The observed increase in ARG richness in the four-plex microbiomes could be attributed to the higher sequencing output in these samples compared to the eight-plex microbiomes, as richness indices are known to be influenced by sequencing depth (Reese and Dunn, 2018) (Figure 3; Supplementary Tables S1). These results align with prior studies showing that sequencing depth has a stronger effect on richness than on diversity indices (Reese and Dunn, 2018; Fernandes et al., 2014). Although few ONT-specific studies have assessed alpha diversity changes at all, and none on multiplexing, our findings support the general observation that richness decreases with reduced per-sample output, while Shannon and Simpson indices remain relatively stable. Finally, recent studies have shown that pig gut microbiome composition and alpha diversity vary with breed, environment, and feed efficiency (Saladrigas-García et al., 2022; Lee et al., 2023; Rahman et al., 2024). In our study, alpha diversity was not significantly different between four- and eight-plex conditions, especially our pigs came from homogeneous background and similar feeding regimes and conditions.

**Figure 4:**
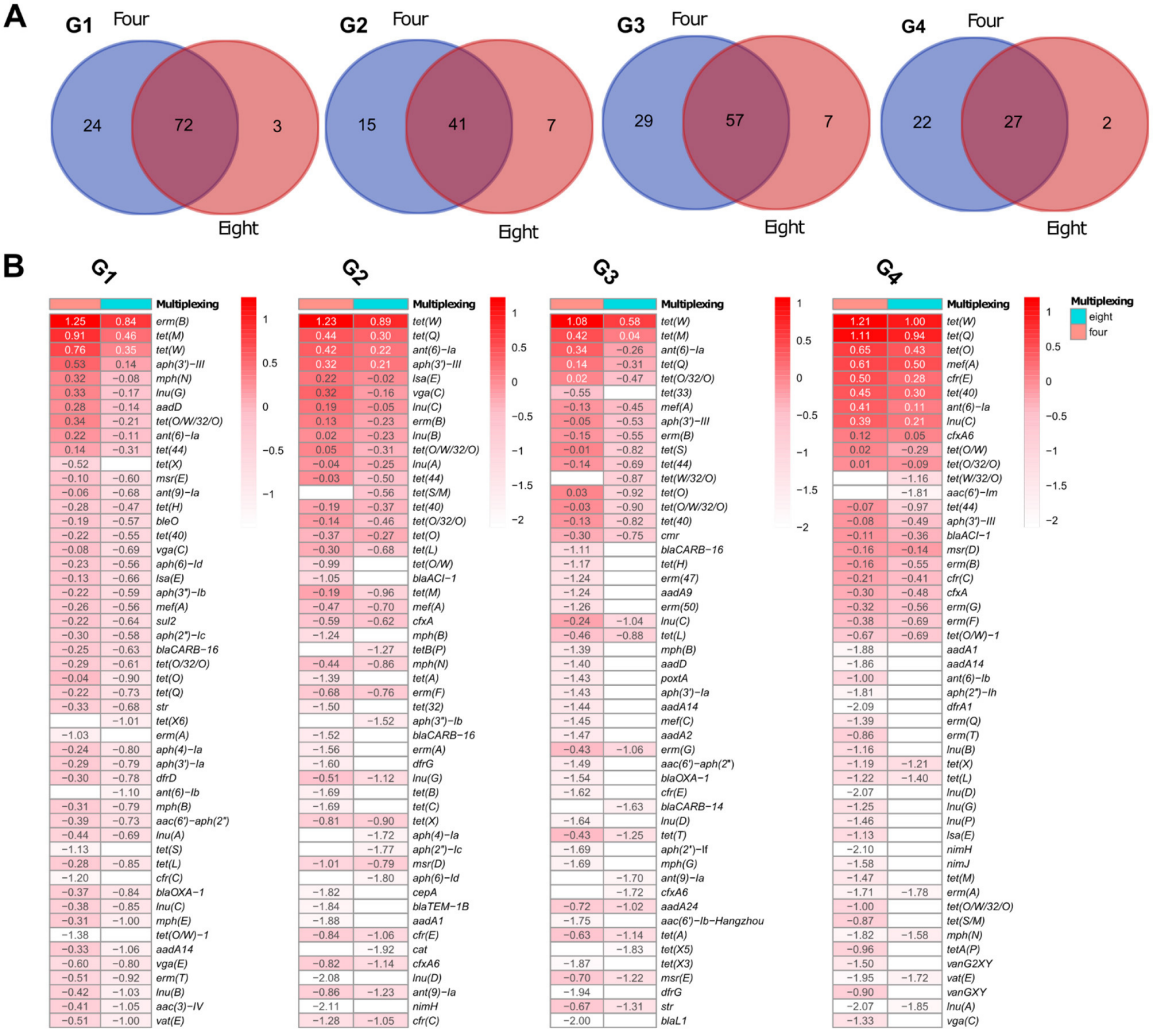
Pig microbiome ARGs from GridION: **(A)** Number of ARGs detected at the two levels of multiplexing, using 0.9 coverage threshold. **(B)** Top 50 ARGs with highest depths (values presented as log10 for visualization) for the pig microbiomes and at the two levels of multiplexing, using 0.9 coverage threshold. The color gradient represents log10 of the depth and the color intensity decreases with decreasing depth. Empty cells represent 0 (log10 of 1).

To evaluate whether sample multiplexing on GridION affects the detection of ARGs, we compared the number and depth of resistance genes identified in pig microbiomes G1 to G4 sequenced at four- and eight-plex levels. Overall, more ARGs were detected in the four-plex condition, but most of these genes were low-depth and the gene difference was not statistically significant (adjusted *p* < 0.05) as estimated by the Kruskal-Wallis test and BH correction (Supplementary S7). Analysis of variance of beta diversity, measured by Bray-Curtis dissimilarities also did not detect significant differences between the identified ARGs in the samples sequenced at four- and eight-plex for both GridION and PromethION (Supplementary Figure S8). Below we summarize the ARG detection patterns across all four samples. On GridION and based on gene coverage threshold at ≥0.9, pig microbiome (G1) had 96 and 75 different observed ARGs at four- and eight-plex, respectively (Supplementary Table S2). In total, 24 ARGs were unique to four-plexed G1 microbiome and three to eight-plexed G1 microbiome (Figure 4A; Supplementary Table S2). Among the 24 ARGs detected at four-plex only, none was of high depth: e.g., *tet*(X) and *erm*(A) were very low in depth (Figure 4B; Supplementary Table S2), and the three ARGs detected at eight were also of low depth: *tet*(X6), *ant*(6)-Ib and *bla*TEM-1B (Figure 4B; Supplementary Table S2). A similar pattern was observed in pig microbiome G2 from GridION, where 15 ARGs (all low depths between −0.99 and −2.11) were detected in the four-plex but not in the eight-plex (Figures 4A, B; Supplementary Table S2). The ARGs detected in the eight-plex but absent in the four-plex (*tet*(S/M), *tet*B(P), *aph*(3′')-Ib, *aph*(4)-Ia, *aph*(2′')-Ic, *aph*(6)-Id, and *cat*) were also of low depth in G2 (Figure 4B; Supplementary Table S2). Similar patterns of low depth ARGs being uniquely detected in either the four- or eight-plex microbiomes were observed in G3 and G4 (details in Supplementary Information Text; Figure 4B; Supplementary Table S2).

To determine whether these observed variations were due to multiplexing level or inherent sequencing variability, we performed triplicate sequencing of the same microbiomes (Full triplicate results in Supplementary Figure S3; Supplementary Table S4). The triplicates showed similar variations, where several low depth ARGs were inconsistently detected across the replicates despite being from the same sample and DNA extract (Supplementary Figure S3; Supplementary Table S4). Specifically, low-abundance ARGs were often missing in one or two triplicates, mirroring the inconsistencies seen between four- and eight-plex multiplexing (Supplementary Figure S3). This suggests that the differences in ARG detection between multiplexing levels are more likely due to technical variability between samples during sequencing, particularly for low-abundance genes, rather than a direct effect of multiplexing, which has been confirmed by our statistical analysis.

**Figure 5:**
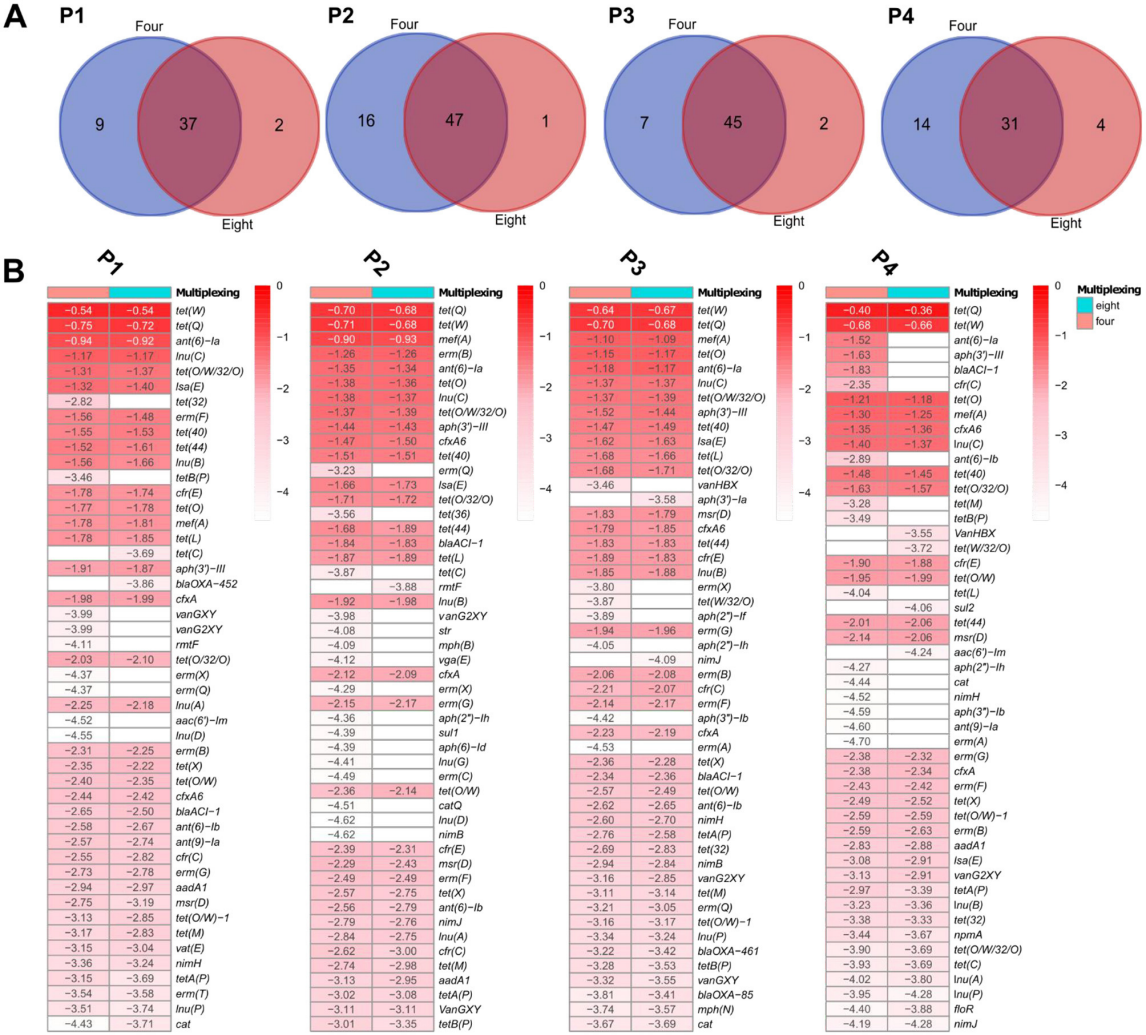
Pig microbiome ARGs from PromethION: **(A)** Number of ARGs detected at the two levels of multiplexing, using 0.9 coverage threshold. **(B)** Top 50 ARGs with highest depths (values presented as log10 for visualization) for the pig microbiomes and at the two levels of multiplexing, using 0.9 coverage threshold.

We next evaluated whether multiplexing affected ARG detection in samples sequenced with PromethION. Similar to GridION, the observed differences between four- and eight-plex were limited to ARGs with low sequencing depth and the differences were statistically not statistically significant (adjusted *p* < 0.05) On PromethION and in microbiome P4, four ARGs detected at four-plex were absent at eight-plex, all with low depths (*ant*(6)-Ia, *aph*(3”)-III, *bla*ACI-1, and *cfr*(C) depth ranging from −1.52 to −2.35) (Figure 5B; Supplementary Table S2X). Four ARGs detected at eight-plex were not found at four-plex, all of which also had low depths (*van*HBX, *tet*(W/32/O), *sul*2, and *aac*(6′)-Im depth from −3.55 to −4.24) (Figures 5A, B). Similar results were recorded from pig microbiomes P1, P2, and P3, where only very few ARGs were detected in eight-plex or four-plex microbiomes only, all with low depths below −3.00 (Figure 5B; Supplementary Table S2), specifically *tet*(C), *bla*OXA-452, *rmt*F, *aph*(3′)-Ia and *nim*J. Therefore here for PromethION, and similar to GridION, both multiplexing levels captured comparable resistome compositions with no statistically significant differences (adjusted *p* < 0.05) in the detected ARGs and the variations were only in the ARGs with low depths.

Similar to AMR in GridION, to validate whether these variations were due to multiplexing level or sequencing variability, we performed triplicate sequencing on PromethION. As observed with GridION, several low-abundance ARGs were inconsistently detected across replicates, despite originating from the same DNA sample (Supplementary Figure S3; Supplementary Table S4). This reinforces that the observed differences in ARG presence between the four- and eight-plex runs are not significant (adjusted *p* < 0.05) and are likely due to stochastic sequencing variation rather than an effect of multiplexing itself. Thus, both platforms demonstrate that multiplexing does not substantially impact resistome composition, with discrepancies primarily occurring for ARGs at the detection limit.

Finally, ALDEx2 tool found no significant differentially abundant ARGs (adjusted *p* < 0.05) between the four- or eight-plex levels of multiplexing on GridION and PromethION (Supplementary Figure S4).

Previous studies have demonstrated that sequencing depth influences the detection of ARGs in metagenomic analyses. For instance, Zaheer et al. (2018) observed that higher sequencing depths led to the identification of a greater number of rare ARGs using Illumina short read sequencing, which were often missed at lower depths. This finding underscores the importance of sufficient sequencing coverage to capture the full spectrum of ARG diversity, particularly those present at low abundance.

### Bacterial community variations across multiplexing levels between microbiomes on GridION and PromethION

Bacterial species and genera richness, similar to the resistomes, were affected by the number of multiplexed samples more than bacterial diversity for both GridION and PromethION (Supplementary Figure S7). The Kruskal-Wallis test followed by BH correction on the first 100 most abundant bacterial species resulted in no significant (adjusted *p* < 0.05) difference between four- and eight-plex for both PromethION and GridION (Supplementary Table S7). Similarly, analysis of variance of beta diversity did not detect significant differences between the identified bacterial species in the samples sequenced at four- and eight-plex for both GridION and PromethION (Supplementary Figure S8).

**Figure 6:**
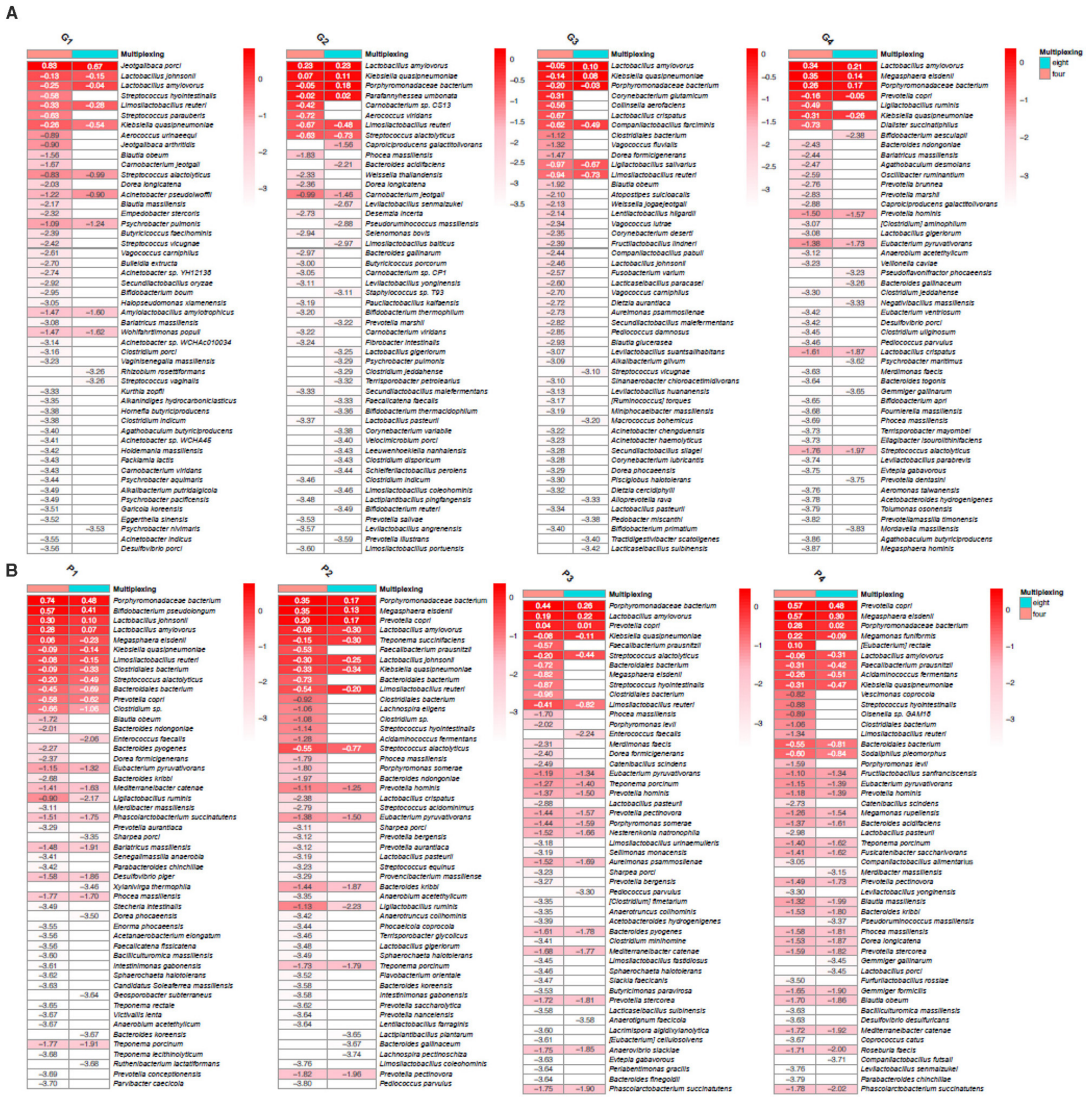
Top 50 most abundant bacterial species (values presented as log10 of the calculated depth for visualization) detected at four- and eight-plex for the pig microbiomes on GridION **(A)** and PromethION **(B)**.

On GridION, for pig microbiome G1, 263, and 171 bacterial species were identified at four- and eight-plex, respectively, with a ≥0.9 coverage threshold (Supplementary Table S3). When comparing four- and eight-plex in the same microbiome, 118 bacterial species were found in four-plex and not in eight-plex and only four of them with relatively higher depths (log10 >-1.00, Supplementary Table S3). In microbiome G2, 83 species were not detected at eight-plex and only two (*Carnobacterium* sp., *Aerococcus viridans*) had high depths (log10 is >-1.00, Figure 6A; Supplementary Table S3). In microbiome G3, 154 species were only detected in four-plex and only three had high depths (log10 >-1.00, Figure 6A; Supplementary Table S3) (*Corynebacterium glutamicum, Collinsella aerofaciens, Lactobacillus crispatus*), while 44 species were not found in four- and found in eight-plex, however, all of them had low depths (below −3.00). In microbiome G4, similar to the other samples, 67 species were unique to four-plex [only two had relatively high depth: *Ligilactobacillus ruminis* and *Dialister succinatiphilus* (−0.49 and −0.73, respectively)] (Figure 6A; Supplementary Table S1) and 27 were unique for eight-plex (all low in abundances below −3.2). The majority of the identified bacterial species were known gut microbiome taxa (e.g., *Bifidobacterium* spp., *Collinsella* spp., *Lactobacillus* spp.) and the variations between the two levels of multiplexing were mostly in taxa of low depth but insignificant (adjusted *p* < 0.05) (Figure 6A; Supplementary Table S3).

On PromethION, microbiome P1 had 320 detected bacterial species at four- and 251 at eight-plex (Supplementary Table S3). In total, 30 bacterial species were undetected at four-plex and all of which had low sequencing depths (log10 below −2.00, Supplementary Table S3), and 99 bacterial species were undetected at eight-plex; with *Blautia obeum*, a widely occurring bacterium in mammal intestines (Liu et al., 2021b), has the highest depth of −1.72 which is still relatively low (Figure 6B; Supplementary Table S3). In microbiome P2, 68% of the species detected at four-plex were also found in eight-plex, three species with greater depth than −1.00, with *Faecalibacterium prausnitzii*, one of the most abundant bacterial species in the gut and associated with healthy gut microbiome (Lopez-Siles et al., 2017), having the highest depth at −0.5 (Figure 6B; Supplementary Table S3). Similarly to the other samples, all undetected species at four-plex had very low abundances (sequencing depths of below −3.5). Of the 92 and 67 undetected species for the P3 and P4 eight-plexed microbiomes, respectively, only *Eubacterium rectale* (microbiome P3), and *Ligilactobacillus ruminis* and *Dialister succinatiphilus* (microbiome P4), all known human and animal gut microbiota species, had sequencing depths >-1.00 (Figure 6B; Supplementary Table S3) (Morotomi et al., 2008; Xiao et al., 2024). Across all samples, bacterial species absent in either multiplexing level were predominantly low in depth and abundance, and species detected exclusively in eight-plex were generally at the very low detection limit (log10 < -3.00). These findings suggest that both four- and eight-plex multiplexing detect comparable bacterial community profiles, with insignificant variations (adjusted *p* < 0.05), mainly affecting low-abundance taxa. Finally, similar to AMR, to validate whether these variations were due to multiplexing or sequencing variability, the triplicate sequencing on GridION and PromethION showed several low-abundance taxa that were inconsistently detected across replicates, despite originating from the same DNA sample (Supplementary Table S5; Supplementary Figure S5). This also confirms that those minor variations are due to sequencing variation rather than an effect of multiplexing itself. Thus, both platforms demonstrate that multiplexing does not substantially impact the bacterial composition.

ALDEx2 tool applying Welch's *t-*test followed byBenjamini-Hochberg correction also concluded that there were no significant differentially abundant species between the samples when sequenced at four- or eight-plex for both GridION and PromethION (Supplementary Figure S4).

### Detection of selected pathogenic species at various levels of multiplexing on GridION and PromethION

We then investigated if selected known pathogenic species were detected at different multiplexing levels on both GridION and PromethION. Detection of pathogenic species in metagenomic samples, especially for surveillance purposes, is important, e.g., to identify causative agents of animal diseases, therefore, it is essential that certain bacterial pathogens (e.g., *Clostridium* spp., *Escherichia coli, Streptococcus* spp.) are detected using metagenomics even when more samples are multiplexed in one library. For this we selected representative bacterial taxa that are known to be involved in animal pathogenicity [adapted from Gand et al., 2024, CDC and WHO], those were counted for 107 bacterial pathogenic species (Supplementary Table S6).

**Figure 7:**
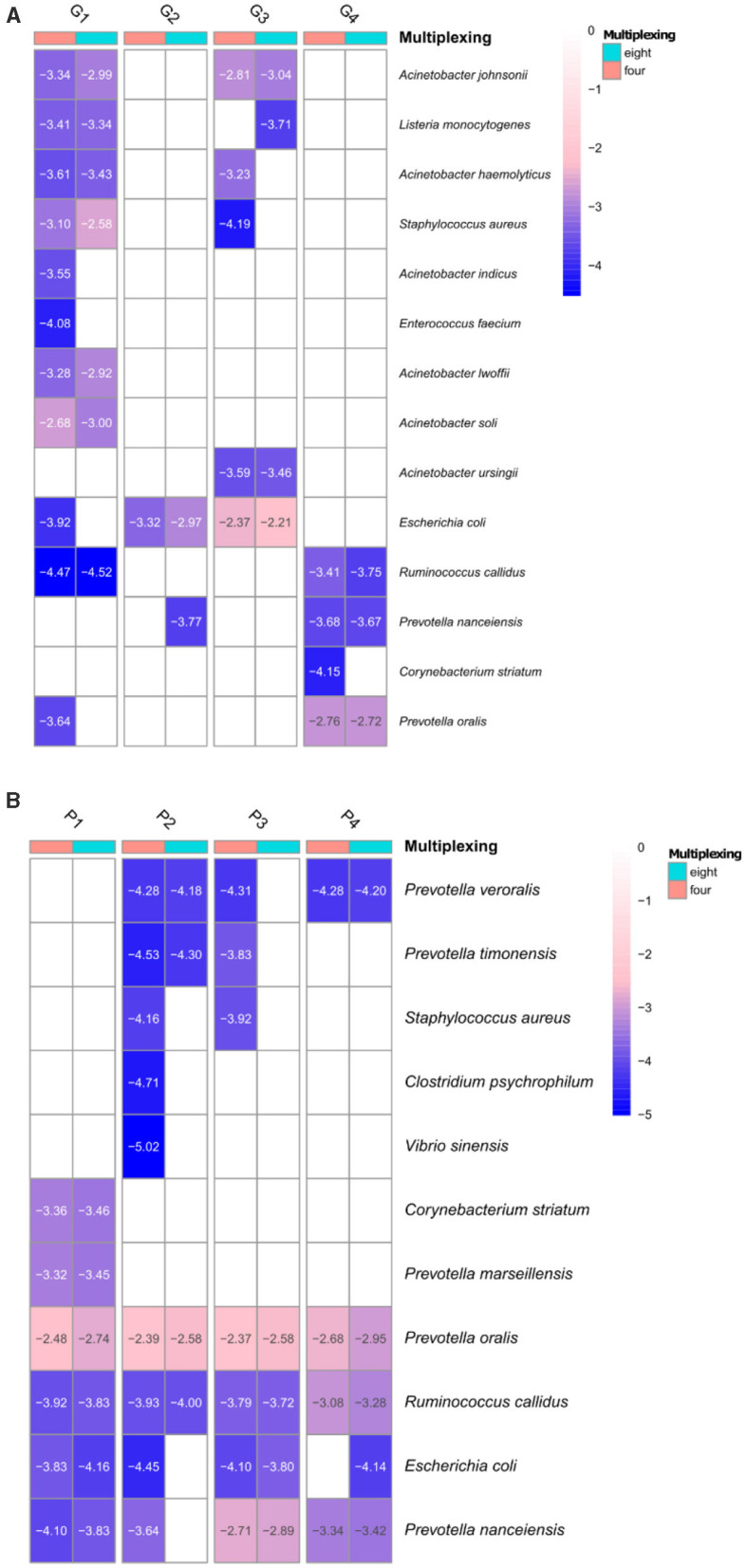
Pathogenic bacterial species detected at the four- and eight-plex on GridION **(A)** and PromethION **(B)** at ≥0.9 coverage threshold of the calculated depths (values are presented as log10 for visualization). Dendrograms are based on Euclidean clustering (≥0.5 coverage threshold of detected pathogens are in Supplementary Figure S6).

We investigated the presence of these bacterial pathogens at ≥ 0.9 coverage threshold (Figure 7 and Supplementary Table S6) for both GridION and PromethION. The detected pathogenic species varied among samples and all of them were present at very low depths in both platforms (Figure 7 and Supplementary Table S6). The majority of the species were detected at both four- and eight-plex and few at four-plex only. Several species, e.g., *Prevotella nanceiensis* from microbiome G2, *Listeria monocytogenes* from G3 and *Escherichia* coli from P4 were detected at eight- but not at four-plex at coverage of ≥0.9, however, all of them were present at low depths (log10 −3.77, −3.71, −4.14, respectively) (Figure 7 and Supplementary Table S6). Similar results were obtained when lower coverage for taxa assignment was applied (≥0.5, details in Supplementary Information Text). However, it has to be noted that none of the samples included in this study originated from diseased pigs (clinical samples), thus the low number and abundance level of the pathogenic bacterial species detected.

## Conclusion

We observed differences in antimicrobial resistance genes (ARGs) and bacterial species between the four-plex and eight-plex sequencing runs on both GridION and PromethION. These differences were primarily observed in low-abundance ARGs and bacterial taxa, where the four-plex identified more genes and species. Despite this, the overall resistome and bacterial community composition remained comparable between the two multiplexing levels on both platforms.

Our results suggest that these variations are more likely due to sequencing variability rather than an effect of increased multiplexing, as similar inconsistencies were observed in triplicate sequencing of the same sample and DNA using the same library preparation methods, particularly in low-abundance ARGs and bacterial taxa. Additionally, while more bacterial pathogens were detected in four-plex than in eight-plex, they were present at very low abundances and depths, suggesting that the overall detection trends remain similar across both multiplexing strategies.

Given that eight-plex sequencing is more cost-effective while still providing comparable resistome and bacterial community profiles, it may be the preferred option, especially with PromethION where large data outputs are obtained, for general detection purposes, especially in large-scale studies or surveillance programs where sequencing costs are a limiting factor. However, for applications requiring highly sensitive pathogen surveillance or detection of rare ARGs, a lower multiplexing level (e.g., four-plex) may be more appropriate to maximize sequencing depth per sample.

These findings contribute to a growing body of work evaluating ONT platforms for microbiome and resistome analyses (Meslier et al., 2022; Gand et al., 2024). While most previous studies have focused on comparisons long-read to short-read sequencing, few have explored how multiplexing affects detection performance on ONT platforms. Our results suggest that careful consideration of sequencing depth is necessary to balance sensitivity and throughput in long-read surveillance applications. This aligns with earlier Illumina-based studies showing that lower sequencing depth reduces the likelihood of detecting low-abundance ARGs, thereby affecting overall resistome richness (Zaheer et al., 2018). However, since multiplexing is rarely addressed in Illumina studies, direct comparisons remain limited. Our study fills a methodological gap by systematically assessing how multiplexing affects detection sensitivity in ONT sequencing—a question that has received limited attention in the literature, despite its growing relevance for large-scale metagenomic studies.

While this study focused on healthy pigs to establish a controlled and diverse baseline for multiplexing comparisons, future studies should apply the same methodology to diseased animals. This would help evaluate how disease-associated microbiome shifts or pathogen overgrowth affect detection sensitivity and multiplexing outcomes.

## Data Availability

The metagenomic sequencing data (FASTQ) have been deposited in the European Nucleotide Archive (ENA) under the project accession number PRJEB86838.

## Supplementary material

Supplementary script 1 Script 1: create_files_from_mapstat_resfinder.py. To concatenate all mapstats and calculate the ARG depths as described in material methods.

Supplementary script 2 create_files_from_mapstat_bacteria.py. To concatenate all mapstats and calculate the bacterial taxa depths as described in material methods.

Supplementary text **More details on resistome variations across multiplexing levels in microbiomes on GridION and PromethION:** Based on the sequencing depth at ≥0.9 gene coverage, pig microbiome (G1) had 96 and 75 observed ARGs at four- and eight-plex, respectively (Supplementary Table S2). 24 genes were unique to four-plexed G1 microbiome and three to eight-plexed G1 microbiome (Figure 4A). Among the 24 genes detected at four-plex only, none was of high sequencing depth: e.g., erm(A) was the highest yet remains very low in depth, cfr(C), sul1, etc. (Figure 4B; Supplementary Table S2), and the three ARGs detected at eight were also of low depth: tet(X6), ant(6)-Ib and blaTEM-1B (Figure 4B; Supplementary Table S2). When also considering the single-plex for microbiome G1, among the top 50 ARGs with highest sequencing depths 10 ARGs detected at single- were not found at four- and eight-plexed microbiomes (Supplementary Table S2). Those genes were floR, tet(O/W/32/O/W/O), tet(32), vanHAX, tet(X6) (at four-plex only), tet(Z), blaDHA-16, tet(31), erm(C) (at eight-plex only) tet(X3), aadA11. Using GridION and fFor pig microbiome G2, fifteen ARGs with sequencing depths between −0.99 and−2.11 were detected in four-plex and not in eight-plex (Figure 4B; Supplementary Table S2), all with relatively low sequencing depth. The ARGs that are detected in eight-plex and not detected at four-plex, were tet(S/M), tetB(P), aph(3′')-Ib, aph(4)-Ia, aph(2′')-Ic, aph(6)-Id and cat (Figure 4B; Supplementary Table SX), all of which are of low sequence depth. Similar pattern of low abundance ARGs were detected in only four-plex or eight-plex in the remaining microbiomes (G3 and G4—further details in Supplementary Information). For microbiome G3, five genes among the top 50 ARGs with highest sequencing depths at eight-plex were not captured by the four-plex multiplexing Twenty-three genes with sequencing depths between −0.55 to −2.00 were not detected at eight-plex, while five genes at eight-plex were not captured by the four-plex sequencing, namely tet(W/32/O), blaCARB-14, ant(9)-Ia, cfxA6 and tet(X5) (Figure 4). Twenty-three genes with sequencing depths between −0.55 to−2.00 were not detected at eight-plex, among them tet(33) with the highest sequencing depth of −0.55 (Figure 4 and Supplementary Table S2). A total of 29 ARGs were detected at eight-plex in microbiome G4 compared to 49 at four-plex. Two of the genes at eight-plex (tet(W/32/O) and aac(6′)-Im) with sequencing depths of −1.16 and −1.81, respectively, were not present at four-plex (Supplementary Table S2). In order to validate if these observed variations were due to the multiplexing or because of inherent variability in sequencing replicates, especially they were all low in depth and abundances, the fecal microbiomes were additionally sequenced in triplicates (Full triplicate results in Supplementary Table S4; and Supplementary Figure S3). Similar variations were observed between the same microbiomes when sequenced in triplicates. I.e., several ARGs were not detected in one or two of the three triplicates even from the same sample and DNA (Supplementary Figure S6). **More details on bacterial community variations across multiplexing levels in microbiomes on GridION and PromethION:** Bacterial species and genera richness, similar to the resistomes, were affected by the number of multiplexed samples more than diversity for both GridION and PromethION P2 Solo (Supplementary Figures S5, S6). On GridION, for pig microbiome G1 at single-plex, 1,559,838 (30.7%) reads were aligned to 426 bacterial species with a threshold of ≥0.9 (Supplementary Table S3), while 592,849 and 203,145 mapped to 263 and 171 bacterial species at four- and at eight-plex, respectively, with a threshold of ≥0.9 (Supplementary Table S3). When comparing four- and eight-plex, 65% of the bacterial species at four- were detected at eight-plex (Supplementary Figure S6; Supplementary Table S3). Deep sequencing (single-plex) of microbiome G1 revealed 195 bacterial species that were not detected at four-plex. Of them, only six had sequencing depths between −0.44 and −0.97 (*Porphyromonadaceae, Olsenella, Megasphaera elsdenii, Aerococcus viridans, Bacteroidales* and *Psychrobacter fulvigenes*), which are mainly marine bacteria (*Psychrobacter fulvigenes*) or part of the indigenous human and animal microbiota (*Porphyromonadaceae, Olsenella, Megasphaera elsdenii* and *Bacteroidales*) and only *Aerococcus viridans*have previously been associated with disease in humans (Mohan, 2017) and pigs (Martín et al., 2007). At eight-plex, 266 bacterial species were not detected at eight-plex compared to single-plex, of them nine with sequencing depths lower than −1.00. Of them, *Jeotgalibaca arthritidis* (−0.97) has been isolated from joint liquids of arthritic pigs (Zamora et al., 2017) and *Streptococcus parauberis* (−0.66), associated with pigs and cows and causing mastitis in cows (Pitkälä et al., 2008). Additionally, 118 bacterial species were found in four and not in eight-plex and only four of them with sequencing depths below −1.00 (described above, Supplementary Table S3). Microbiome G2, 83 species were not detected at eight-plex and only two (*Carnobacterium* sp., *Aerococcus viridans*) had sequencing depths below −1.00. In microbiome G3, 154 species less were detected in eight-plex and only three of them with sequencing depths below −1.00 (*Corynebacterium glutamicum, Collinsella aerofaciens, Lactobacillus crispatus*), while 44 species were not found in four- and found in eight-plex, however, all of them had low sequencing depths (below −3.00). In microbiome G4, 224 species were present in four and 184 in eight-plex. Similarly to the other samples, 67 species were unique for four-plex (only 2 in *Ligilactobacillus ruminis* and *Dialister succinatiphilus* (−0.49 and −0.73) and 27 were unique for eight-plex (all below −3.2) and only *Bifidobacterium aesculapii* was detected in eight but not in four-plex with seq depth of −2.4. **More details on pathogenic bacterial detection using different levels of multiplexing:** At the less strict threshold of coverage (≥0.5), on GridION four species (*Myroides injenensis, Prevotella nanceiensis, Klebsiella oxytoca* at −2.83 and *Prevotella marseillensis*) were detected at eight-plex but not at four, while no species were detected at eight- but not at four-plex for PromethION P2 Solo (Supplementary Table S6). out of place: Variations in pathogenic bacterial detection between the microbiomes because of the number of, multiplexedsequenced samples in a flowcell could have great implications on pathogen surveillance. For example, *C. perfringens* type C and hemolytic *E. coli* cause hemorrhagic gastroenteritis and death in piglets (47). Healthy animals, including sows, may variably carry small numbers of *C. perfringens* type C in their intestines, which could proliferate rapidly (47), therefore detection of even low pathogen numbers is crucial for disease prevention and surveillance. **Testing the reproducibility of repeated sequencing in detecting ARGs and bacteria on GridION and PromethION P2 Solo** To assess if repeated sequencing of a metagenomic sample can detect same ARGs and bacterial species, four samples (P1, P2, P3, and P4) were sequenced on both GridION and PromethION P2 Solo in triplicate each at four- and eight-plex. The detected ARGs across the three replicates varied for each sample, however, the most abundant ARGs (with highest sequencing depths) were captured in all three replicates sequenced on GridION and PromethION P2 Solo at both levels of multiplexing. Similarly, for bacterial species, with some exceptions, the most abundant bacteria were detected in all three replicates for both levels of multiplexing. The least abundant bacteria were identified at four- but not at eight-plex (Supplementary Tables S4, S5).

Supplementary Figure S1 Total number of high quality reads for one, four and eight samples per flowcell sequenced on GridION and four and eight samples per flowcell sequenced on PromethION P2 Solo.

Supplementary Figure S2 Average number of high quality base-pairs for four and eight samples per flowcell sequenced on GridION and PromethION P2 Solo.

Supplementary Figure S3 Top 50 (or all ARGs, if their total number in a sample was < 50) ARGs with highest sequencing depths across the three replicates for the four samples sequenced at four- and eight-plex on GridION **(A)** and PromethION P2 Solo **(B)**.

Supplementary Figure S4 Differential abundance effect plot showing the within-group dispersion of depth values for bacterial species and ARGs between four- and eight-plex runs for both GridION **(A)** and PromethION P2 Solo **(B)**. Each point represents a feature (ARG or taxon), with lighter or darker shades of gray reflecting different levels of within-group variance as calculated by ALDEx2. Statistically significant features (adjusted *p* < 0.05, Benjamini–Hochberg corrected) would appear in red (none were found in our case). The gray dotted vertical line indicates an effect size of 1.

Supplementary Figure S5 Top 50 bacterial species with highest sequencing depths across the three replicates for the four samples sequenced at four- and eight-plex on GridION **(A)** and PromethION P2 Solo **(B)** at a sequencing depth threshold of 0.9.

Supplementary Figure S6 Pathogenic bacterial species detected at the four- and eight-plex on GridION **(A)** and PromethION P2 Solo **(B)** at coverage of ≥0.9 and ≥0.5 as threshold for calculation of depths.

Supplementary Figure S7 Chao1 richness index and Shannon and Simpson diversity indices calculated from the abundance tables at species level for GridION **(A)** and PromethION P2 Solo **(B)**.

Supplementary Figure S8 Principal Coordinates Analysis (PCA) of beta diversity based on Bray–Curtis dissimilarities of antimicrobial resistance gene (ARG) composition across four- and eight-plex sequencing conditions. Samples were sequenced using GridION and PromethION. Each point represents a sample, colored by the multiplexing level.

Supplementary Table S1 All run raw outputs in read counts and in basepair counts.

Supplementary Table S2 List of all identified ARGs in the original samples with four- and eight-plex of the pig microbiomes on both GridION and PromethION in the original setup of metagenomes in Figure 1. Each sheet represents one run (G, GridION - P, PromethION).

Supplementary Table S3 List of all identified bacterial species in the original samples with four- and eight-plex of the pig microbiomes on both GridION and PromethION in the original setup of metagenomes in Figure 1. Each sheet represents one run (G: GridION - P, PromethION).

Supplementary Table S4 List of all identified ARGs in the triplicate samples with four- and eight-plex of the pig microbiomes on both GridION and PromethION. Those are only for the triplicate samples to confirm the variations observed between sequenced samples. Each sheet represents one run and one multiplexing level (G: GridION - P, PromethION).

Supplementary Table S5 List of all identified bacterial species in the triplicate samples with four- and eight-plex of the pig microbiomes on both GridION and PromethION. Those are only for the triplicate samples to confirm the variations observed between sequenced samples. Each sheet represents one run and one multiplexing level (G: GridION - P, PromethION).

Supplementary Table S6 List of the pathogenic bacterial species detected at the four- and eight-plex on GridION (sheets named G) and PromethION (sheets named P) at coverage of ≥ 0.9 (G_0.9 and P_0.9) and ≥ 0.5 (G_0.5 and P_0.5) as threshold for calculation of depths. Last sheet has the full list of all the potential pathogenic species we investigated their presence.

Supplementary Table S7 Results of Kruskal–Wallis tests comparing the abundance of antimicrobial resistance genes (ARGs) and bacterial species between four- and eight-plex sequencing runs on GridION and PromethION platforms. Analyses were performed separately for each platform. *P-*values were corrected using the Benjamini–Hochberg procedure to control the false discovery rate (FDR). No features were found to be significantly different (adjusted *p* < 0.05) between multiplexing levels. Each Sheet is a platform and a feature (either bacteria or ARG).
