# Supplementary figures and images for: Impact of sample multiplexing on detection of bacteria and antimicrobial resistance genes in pig microbiomes using long-read sequencing
Source: Front Microbiol. 2025 Jun 19;16:1597804. doi: 10.3389/fmicb.2025.1597804 (PMC12222346; doi:10.3389/fmicb.2025.1597804)

Figure S1:


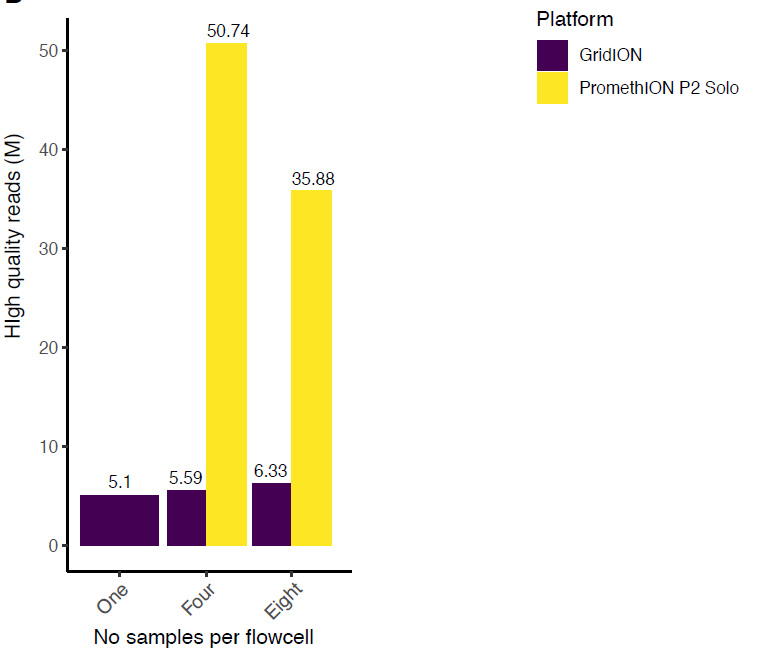


Figure S2:


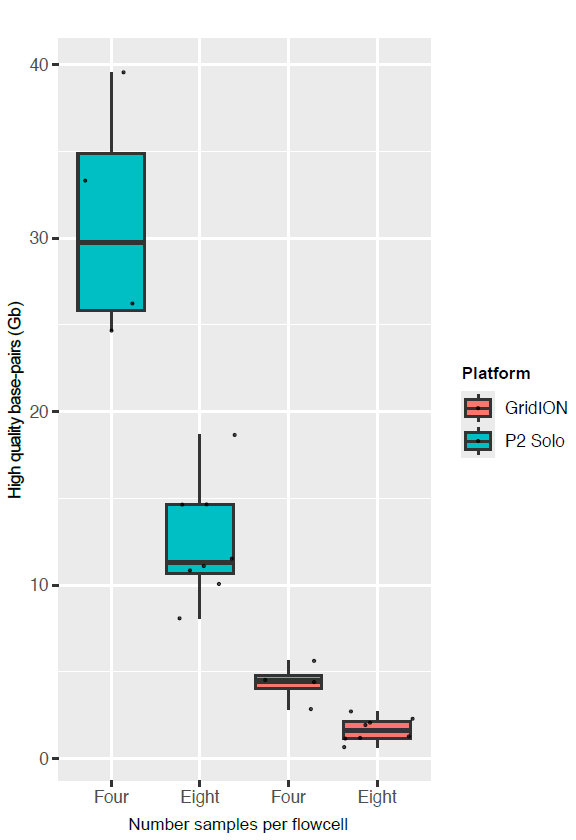


Figure S3:


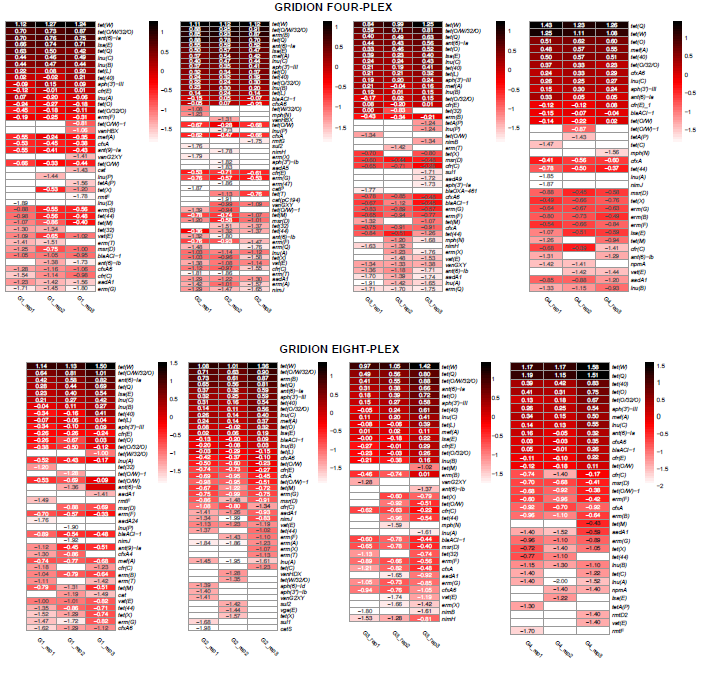


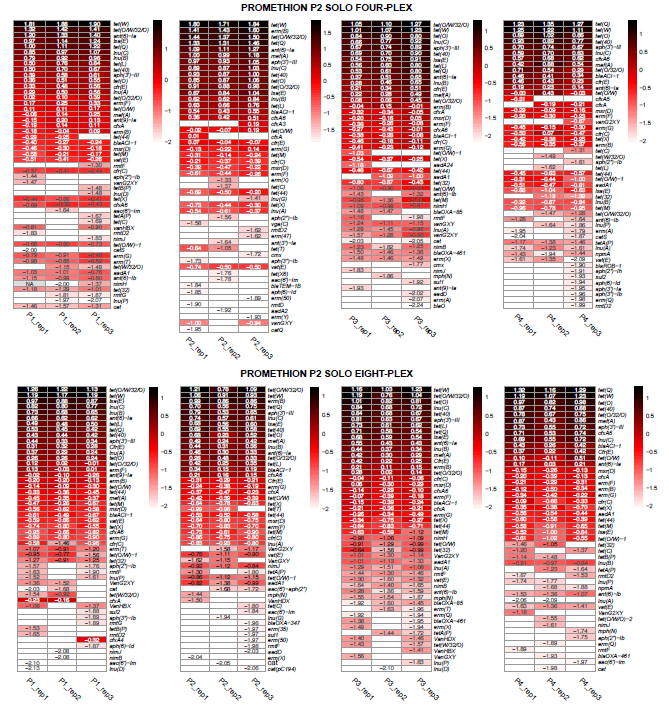


Figure S4:


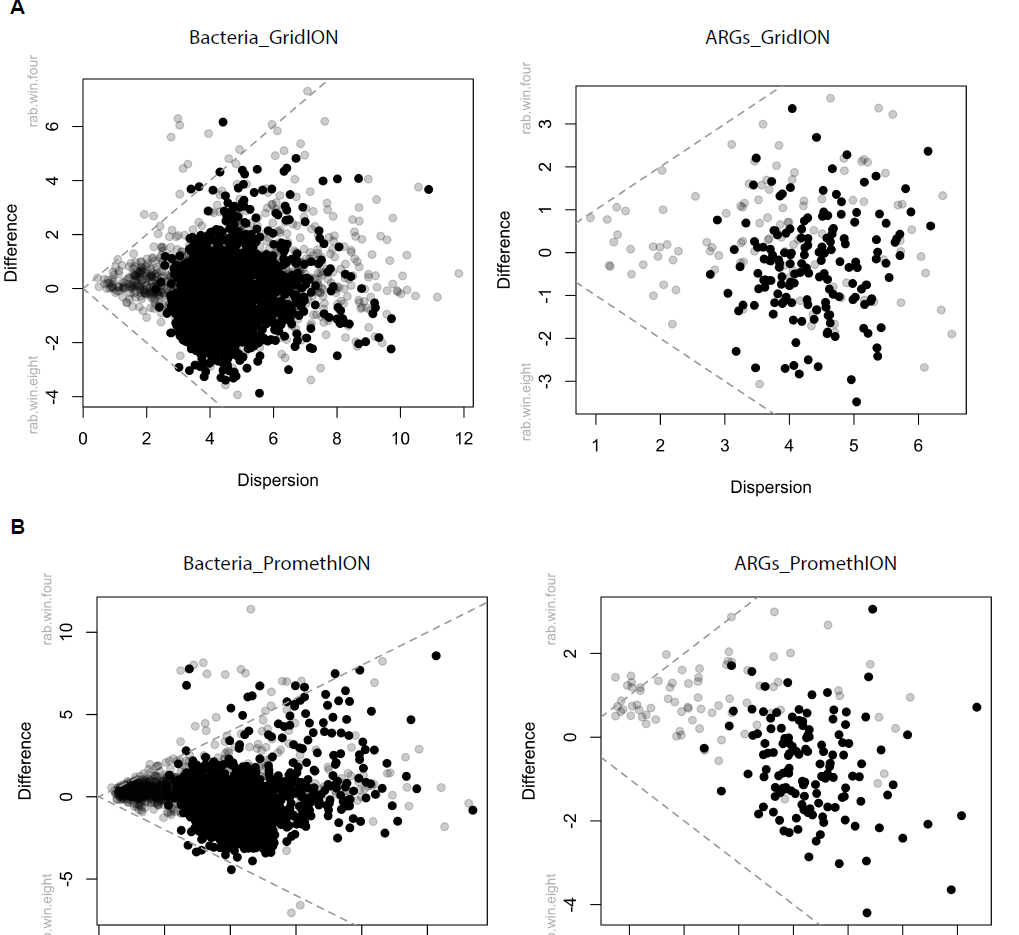


Figure S5:


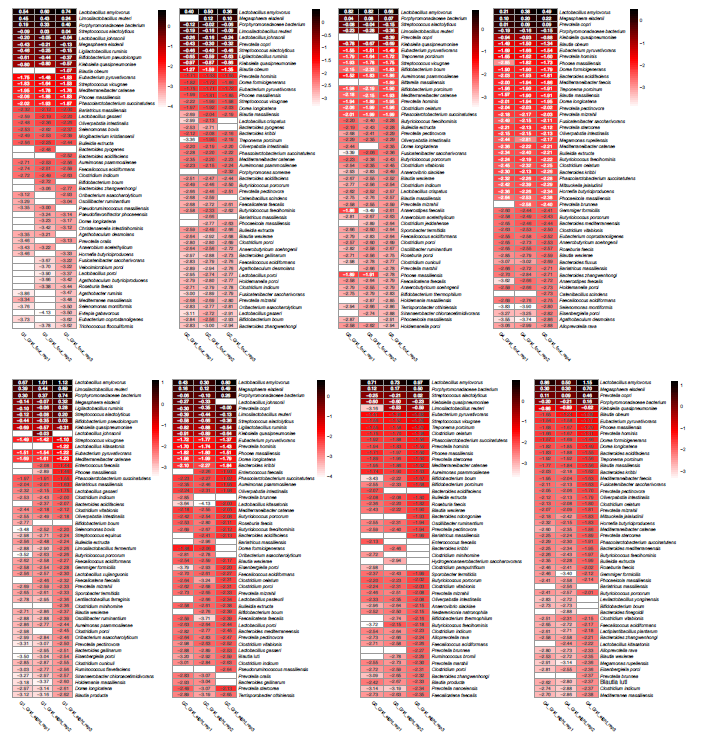


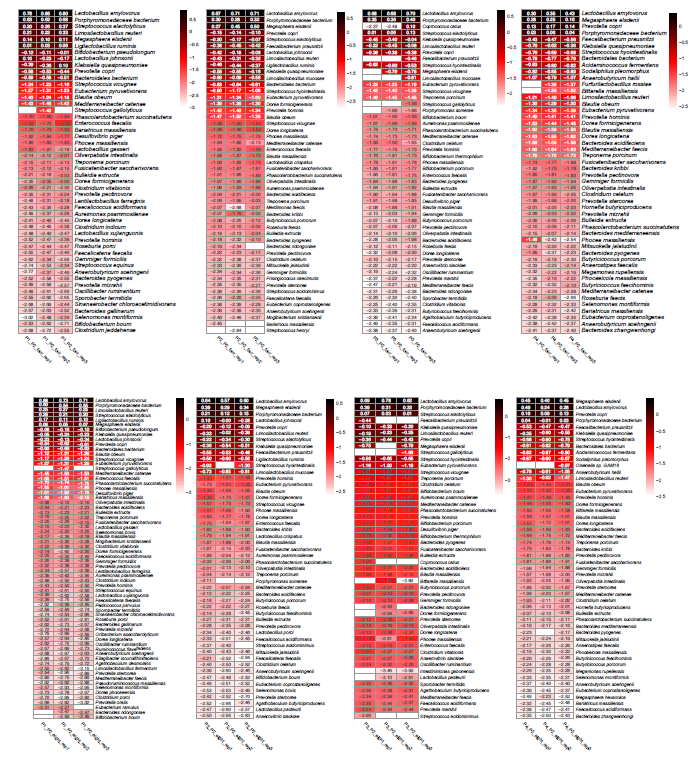


Figure S6:


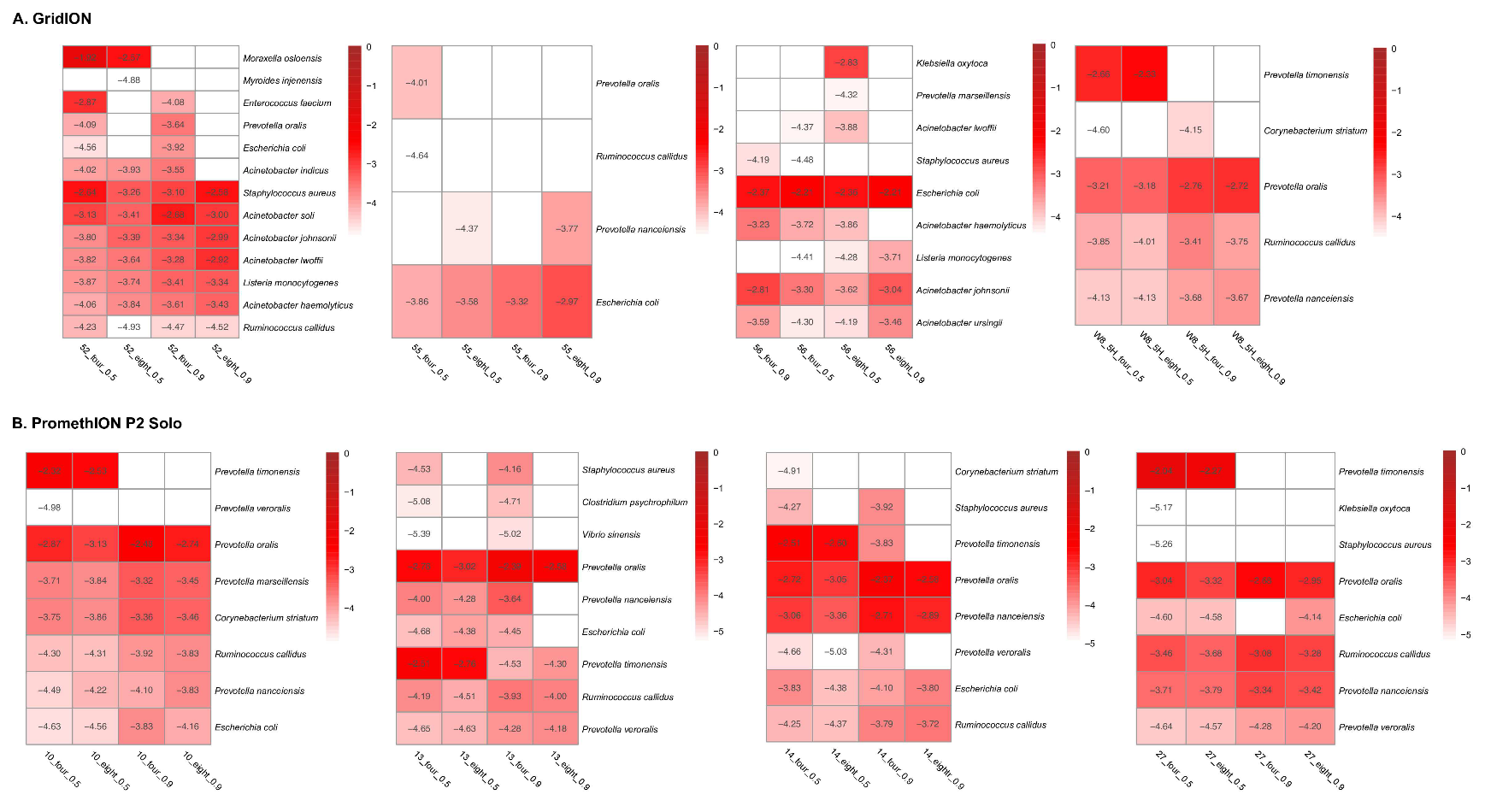


Figure S7:


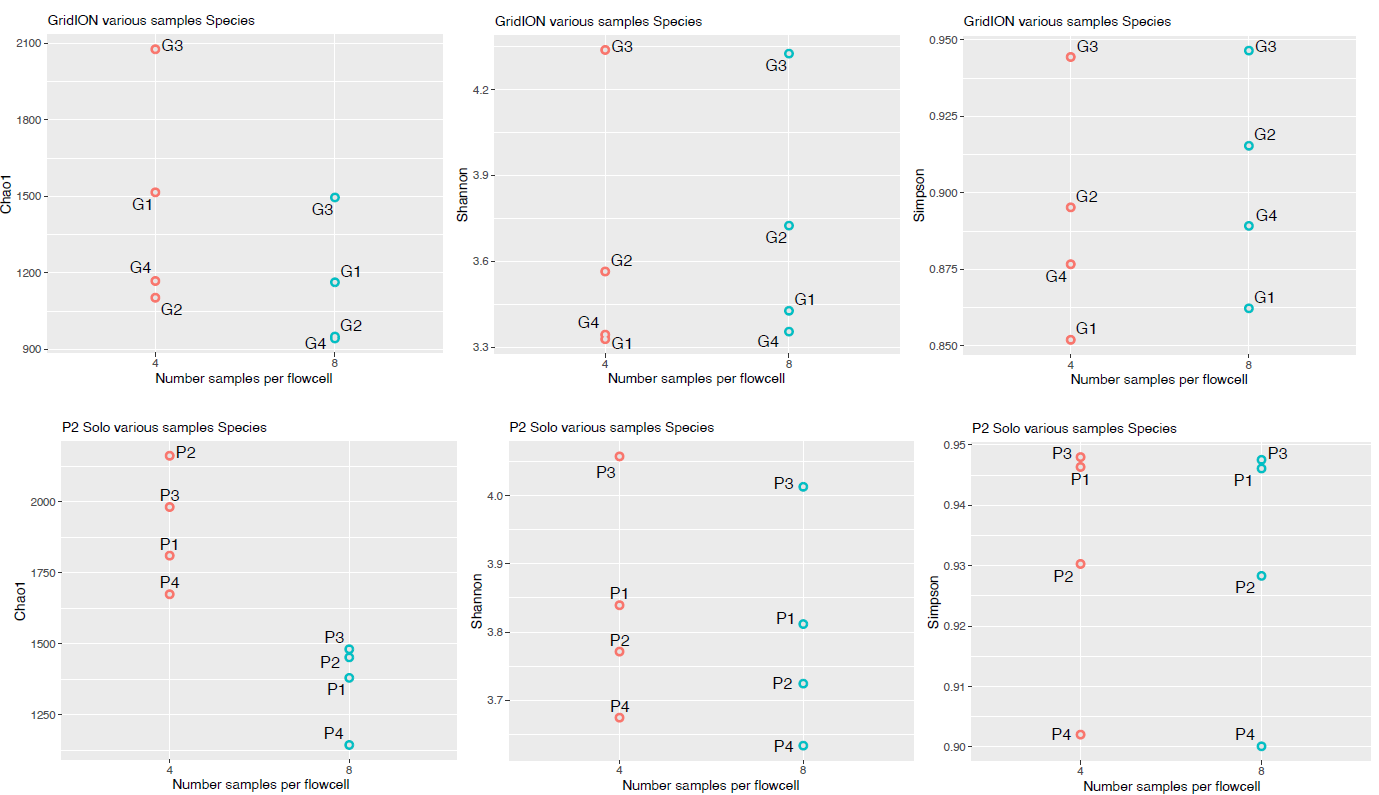


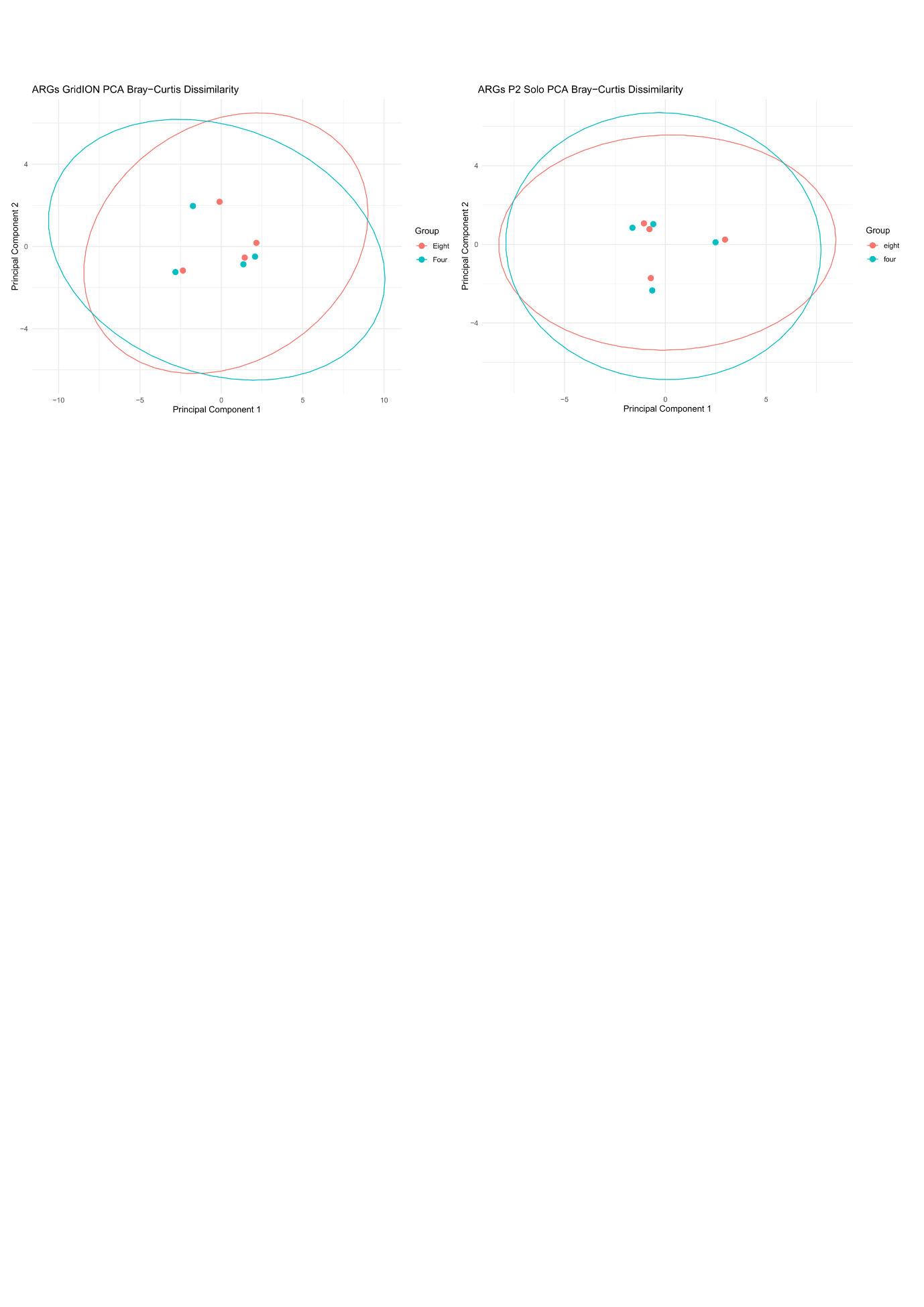
Figure S8:

Supplement: Supplementary script 1 — Script 1: create_files_from_mapstat_resfinder.py. To concatenate all mapstats and calculate the ARG depths as described in material methods. [file Data_Sheet_1.docx]
